# Disrupting PTPRJ transmembrane-mediated oligomerization counteracts oncogenic receptor tyrosine kinase FLT3 ITD

**DOI:** 10.3389/fonc.2022.1017947

**Published:** 2022-11-14

**Authors:** Marie Schwarz, Sophie Rizzo, Walter Espinoza Paz, Anne Kresinsky, Damien Thévenin, Jörg P. Müller

**Affiliations:** ^1^ Institute for Molecular Cell Biology, CMB - Center for Molecular Biomedicine, University Hospital Jena, Jena, Germany; ^2^ Department of Chemistry, Lehigh University, Bethlehem, PA, United States; ^3^ Leibniz Institute on Aging, Fritz Lipmann Institute, Jena, Germany

**Keywords:** transmembrane domain oligomerization, receptor protein tyrosine phosphatase (RPTP), DEP-1, receptor tyrosine kinase (RTK), oncogenic FLT3 ITD, signal transduction, AML

## Abstract

The receptor protein tyrosine phosphatase (RPTP) PTPRJ (also known as DEP-1) has been identified as a negative regulator of the receptor tyrosine kinase FLT3 signalling *in vitro*. The inactivation of the *PTPRJ* gene in mice expressing the constitutively active, oncogenic receptor tyrosine kinase FLT3 ITD aggravated known features of leukaemogenesis, revealing PTPRJ’s antagonistic role. FLT3 ITD mutations resulting in constitutively kinase activity and cell transformation frequently occur in patients with acute myeloid leukaemia (AML). Thus, *in situ* activation of PTPRJ could be used to abrogate oncogenic FLT3 signalling. The activity of PTPRJ is suppressed by homodimerization, which is mediated by transmembrane domain (TMD) interactions. Specific Glycine-to-Leucine mutations in the TMD disrupt oligomerization and inhibit the Epidermal Growth Factor Receptor (EGFR) and EGFR-driven cancer cell phenotypes. To study the effects of PTPRJ TMD mutant proteins on FLT3 ITD activity in cell lines, endogenous PTPRJ was inactivated and replaced by stable expression of PTPRJ TMD mutants. Autophosphorylation of wild-type and ITD-mutated FLT3 was diminished in AML cell lines expressing the PTPRJ TMD mutants compared to wild-type-expressing cells. This was accompanied by reduced FLT3-mediated global protein tyrosine phosphorylation and downstream signalling. Further, PTPRJ TMD mutant proteins impaired the proliferation and *in vitro* transformation of leukemic cells. Although PTPRJ’s TMD mutant proteins showed impaired self-association, the specific phosphatase activity of immunoprecipitated proteins remained unchanged. In conclusion, this study demonstrates that the destabilization of PTPRJ TMD–mediated self-association increases the activity of PTPRJ *in situ* and impairs FLT3 activity and FLT3-driven cell phenotypes of AML cells. Thus, disrupting the oligomerization of PTPRJ *in situ* could prove a valuable therapeutic strategy to restrict oncogenic FLT3 activity in leukemic cells.

## Introduction

The receptor tyrosine kinase (RTK) FLT3 (fms-like tyrosine kinase 3) is predominantly expressed in early hematopoietic progenitor cells of both myeloid and lymphoid lineage (1–3). and is activated by its ligand FL. Wild-type (wt) FLT3 is present at the plasma membrane in the form of inactive monomers, whose auto-inhibited conformation is maintained by interactions between the juxtamembrane (JM) and kinase domains (4, 5). Binding of homo-dimeric FL induces receptor dimerization and trans-autophosphorylation on intracellular tyrosine residues (6, 7), The ligand-induced receptor phosphorylation results in the recruitment of adapter and scaffolding proteins that mediate the activation of phosphatidylinositol 3-kinase (PI3K)/protein kinase B (AKT) and rapidly accelerated fibrosarcoma (RAF)/mitogen-activated protein kinase (MAPK) signalling pathways (4, 8–10).

Activating mutations of FLT3 occur in approximately 25 – 30% of acute myeloid leukaemia (AML) patients. Internal tandem duplications (ITD) within the FLT3 JM domain constitute the most prevalent type of mutation and are associated with a poor patient prognosis. ITD mutations are characterized as in-frame duplications in the region encoding the receptor’s JM domain (11–13). As a consequence, FLT3 ITD mutations presumably disrupt the auto-inhibitory activity of the JM domain (4, 5), leading to ligand-independent dimerization, auto-phosphorylation, and constitutive activation of the receptor (10, 11, 14). In contrast to the wt receptor and due to its constitutive kinase activity, ITD-mutated FLT3 is predominantly retained in the endoplasmic reticulum (ER) and Golgi compartments (15–17), from where it strongly promotes activation of signal transducer and activator of transcription 5 (STAT5) (15–17). Due to a residual pool of FLT3 ITD reaching the plasma membrane, constitutive activation of AKT and extracellular signal-regulated kinases 1/2 (ERK1/2) signalling can also be found (16, 18, 19).

FLT3 ITD promotes factor-independent growth and blocks the differentiation of myeloblastic cells *in vitro (* 9, 10, 20 *).* Moreover, it has been shown to induce leukemic cell transformation *in vitro* (10) and to drive the developmentof myeloproliferative disease in murine bone marrow transplantation models (14, 21). Furthermore, FLT3 ITD induces the production of high levels of reactive oxygen species (ROS) *via* STAT5-mediated activation and enhanced expression of NADPH oxidase (NOX) 4 (22, 23). This contributes to FLT3 ITD–driven cellular proliferation and transformation by several mechanisms, including the oxidative inactivation of the antagonistic PTP DEP-1 (22, 24, 25).

Protein tyrosine phosphatases (PTPs) are important modulators of reversible protein tyrosine phosphorylation, counterbalancing the action of protein tyrosine kinases (26, 27). PTPRJ, also known as DEP-1, CD148 or RPTPη, is an RPTP encoded by the *PTPRJ* gene. It is composed of a single PTP domain, a transmembrane segment, and an extracellular domain that contains eight fibronectin type III repeats, and the mature protein is highly N-glycosylated (28, 29).

PTPRJ is an essential negative regulator of growth factor signalling and, therefore, of cell proliferation and migration processes (30–33). For instance, PTPRJ restricts the activity of the platelet-derived growth factor β receptor (30, 31, 34), vascular endothelial growth factor receptor (32, 35) hepatocyte growth factor receptor MET (33, 36), epidermal growth factor receptor (EGFR) (37, 38), and FLT3 (39, 40). This highlights the role of PTPRJ as a candidate tumor-suppressor, especially in the context of RTK-driven cancers (41, 42). For example, PTPRJ has been shown to suppress the proliferation of glioblastoma cells by attenuating EGFR signalling and internalization (37). Furthermore, PTPRJ deficiency has been implied to promote meningioma cell motility and invasiveness (31, 43, 44) and to contribute to FLT3 ITD–driven leukemic cell transformation (24).

Besides indirect mechanisms such as modulation of expression levels or subcellular distribution, the specific activity of PTPs can be regulated by ligand binding, oxidation, or dimerization (27, 45). Binding of ligands to the extracellular domains of RPTPs has been shown to modulate specific PTP activity. PTPRJ can be activated by TSP1, but it is mechanistically not understood (46, 47). On the contrary, reactive oxygen species (ROS) can reversibly oxidize the catalytic cysteine of PTPs, leading to the formation of sulfenic acid derivates, sulfenylamides, or disulfides and thereby inactivating the PTP (48–50). Upon stimulation, some RTKs induce the production of ROS and thus transiently inhibit antagonistic PTPs from allowing for efficient signal propagation (45). In particular, pathological ROS production by oncogenic RTKs can remove regulatory constraints from counteracting PTPs through this mechanism (22, 49, 51) as seen for the ROS-mediated inactivation of PTPRJ in FLT3 ITD–positive leukemic cells (22, 24).

For some RPTPs, including RPTPα, SAP1, and PTPRJ, TMD-mediated interactions have been demonstrated to contribute to dimerization (52–54). Homodimerization of the RPTP at the plasma membrane is typically associated with inactivation of the PTP (52, 53, 55) due to blocked access to the catalytic site (45, 56, 57). In mutational studies of PTPRJ TMD, the residues G979, G983, and G987 have been identified as major mediators of self-association (38, 54) through a specific contact interface consistent with a double GxxxG zipper motif. In accordance with a dimerization-mediated suppression of PTP activity, disruption of those PTPRJ TMD interactions by introducing G-to-L mutations was shown to enhance PTPRJ association with and dephosphorylation of EGFR (38). This is supported by structural analyses on the PTPRJ rat homolog that suggest that a wedge-like segment would block substrate access to the catalytic site in dimers, similar to RPTP*α* D1 (58).

Given the tumor-suppressing function of PTPs such as PTPRJ, strategies to enhance PTP activity have been developed by exploiting their regulatory mechanisms, such as ligand binding or oligomerization (26, 59). Given the enhanced activity of PTPRJ following stimulation with its ligand TSP1 (60, 61), PTPRJ–activating therapies may be developed by targeting the PTPRJ ectodomain, for example, with monoclonal antibodies (33) or peptide agonists (62, 63). Based on the structure-function relationship of PTPRJ TMD–mediated oligomerization described above, Bloch et al. designed agonistic peptides that bind to PTPRJ TMD and disrupt it its interactions (38). *In vitro* treatment of EGFR-driven cancer cells with those peptide agonists was shown to decrease PTPRJ self-association, enhance its activity on EGFR, and ultimately inhibit EGFR-driven cancer cell migration (38).

Several PTPs are known to be involved in the regulation of FLT3 signalling. SHP2 has been shown to positively regulate ERK1/2 and STAT5 activation downstream of FLT3 (7, 64, 65). In contrast, other phosphatases, including SHP1, PTP1B, and CD45, have been found to restrict FLT3 activity (66, 67). PTPRJ was first characterized as an antagonist of FLT3 in an shRNA-based screen to identify PTPs regulating FLT3 activity. Depletion of PTPRJ in THP-1 and 32D cells expressing wt FLT3 was demonstrated to enhance FL-induced FLT3 autophosphorylation and ERK1/2 signalling, ultimately stimulating proliferation and clonal growth (39). FLT3 was also established as a *bona fide* substrate of PTPRJ based on *in vitro* dephosphorylation of immunoprecipitated FLT3 by recombinant PTPRJ and direct interaction evidenced by co-immunoprecipitation and *in situ* proximity ligation assay (PLA). These studies also indicated that PTPRJ could play a role in suppressing both basal and ligand-induced activity of FLT3 (39, 40).

Here we show that despite disruption of PTPRJ oligomerization of PTPRJ Gly-to-Leu TMD mutations at positions G979, G983, and G987 specific phosphatase activity of immunoprecipitated PTPRJ proteins was not altered. Stable expression of PTPRJ TMD mutants in leukemic cells inactivated for their endogenous PTPRJ expression resulted in diminished phosphorylation of wt and ITD-mutated FLT3 compared to cells expressing wt PTPRJ. This was accompanied by reduced FLT3-mediated global protein tyrosine phosphorylation and FLT3-mediated downstream signalling. Lastly, PTPRJ TMD mutant proteins impaired the proliferation and *in vitro* transformation of leukemic cells.

## Material and methods

### Cell lines, cytokines, and antibodies

The human leukemic cell lines THP-1 (expressing wt FLT3) and MV4-11 (expressing FLT3 ITD) were cultured in RPMI-1640 supplemented with stabilized glutamine, 10% heat-inactivated FCS. The IL-3-dependent murine myeloid cell line 32D clone 3 (32D) (German Collection of Microorganisms and Cell Cultures (DSMZ), Braunschweig, Germany) was maintained in RPMI 1640 medium supplemented with sodium pyruvate (5 mg/ml), 10% heat-inactivated fetal calf serum (FCS), L-glutamine (2 mM), and IL-3 (1 ng/ml). 32D cells stably expressing FLT3 ITD were kindly provided by Drs. R. Grundler and J. Duyster (Technical University Munich, Germany) (14). All cell lines were inactivated for endogenous PTPRJ production using CRISPR/Cas9-mediated gene inactivation. Biallelic gene disruption, further described as PTPRJ KO, was confirmed by sequencing and immunologically. The human embryonal kidney cell line HEK293T (obtained from the DSMZ, Braunschweig, Germany) was cultured in DMEM/F12 supplemented with stabilized glutamine, 10% fetal calf serum (FCS) (Invitrogen, Darmstadt, Germany). Cells were cultured in a humidified incubator at 37°C with 5% CO_2_.

Recombinant human FL and murine IL-3 were purchased from PeproTech Ltd., London, UK.

Anti-P-AKT (Ser-473) (catalogue no. 9271), anti-P-p44/42 MAPK (Thr-202/Tyr-204) (catalogue no. 9106), anti-ERK1/2 (catalogue no. 9107) anti- STAT5 (catalogue no. 9310) and anti-P-FLT3 Y591 (3461)) were from Cell Signalling Technology (Frankfurt, Germany). Anti-AKT (sc-8312) and anti-STAT5 (sc-835) were purchased from Santa Cruz, and anti-P-STAT5 (ab32364) was from Abcam. Polyclonal anti-FLT3 antibody (from goat, AF768) recognizing the extracellular domain of the murine protein, anti-DEP-1 antibody (from goat, AF1934) recognizing murine DEP-1 and cross-reacting with human DEP-1 were obtained from R&D Systems (Wiesbaden, Germany). ß-actin antibody (A5441) was from Sigma-Aldrich. Antibodies recognizing phosphotyrosine (05-321) were purchased from Upstate Biotechnology, Inc. (Milton Keynes, UK). Human FLT3 was detected with polyclonal rabbit antibodies, kindly provided by Lars Rönnstrand (Lund, Sweden). Immune detection of HA-tagged DEP-1 was done with antibody 3724 from Cell Signaling Technology (Leiden, The Netherlands). Antibodies recognizing ß-actin or vinculin were obtained from Sigma. HRP-coupled secondary anti-mouse IgG and anti-rabbit IgG antibodies were from KPL (Gaithersburg, MD). HRP-coupled secondary anti-goat IgG (sc2056) was from Santa Cruz Biotechnology. HA-tagged DEP-1 proteins were immunoprecipitated using an α-HA-tag antibody (kindly provided by Sebastian Drube, Institute of Immunology, University Hospital Jena).

### DNA expression vectors

Retroviral pBABE vectors expressing human PTPRJ wt or G-L TMD mutants were published earlier (38). For efficient immunoprecipitation 3’-end of the gene was replaced by a human hemagglutinin (HA) tag derived from pcDNA3.1-PTPRJ-HA (our laboratory collection). Obtained plasmids were sequence validated.

### Production of pseudoviral particles, cell transduction, and cell establishment

For the generation of retroviral particles, HEK293T cells were transfected with pBABE plasmids encoding PTPRJ and pVSVg and pGag/Pol packaging vectors using polyethylenimine (PEI). Retroviral particles were collected 24, 48, and 72 h post-transfection. Cell-free culture supernatants were concentrated using Amicon Ultra-15 Centrifugal Filter Units (Millipore, # UFC903024). THP-1, MV4-11, or 32D FLT3 ITD PTPRJ KO cells were infected three times with the pseudotyped particles in the presence of 8 mg/ml polybrene (1,5-dimethyl-1,5-diazaundecamethylene polymethobromide, AL-118, #10,768-9, Sigma-Aldrich). Cell lines were cultivated for at least 3 weeks post-transduction.

To enrich cells re-expressing PTPRJ, transduced cells were stained with APC-coupled anti-PTPRJ (CD148) antibody and subsequently purified by flow cytometric cell sorting according to their PTPRJ surface levels. APC signal was measured at 633/660 nm ex/em using a BD FACS Canto and FACS Diva v7.0 software (BD). Data were analyzed in FlowJo v10.8.0 (BD). Similar PTPRJ levels were monitored using immunoblotting. Not all cell populations could be sorted to an equivalent PTPRJ level. Thus, only data from cells with comparable PTPRJ levels were used here.

### Analysis of signal transduction

For analysis of cell signalling, cells were starved for 4 hours. FLT3 ITD–expressing cell lines 32D FLT3 ITD, and MV4-11 were treated with 0.5 μM of the NAD(P)H oxidase inhibitor diphenyleneiodonium (DPI) or mock (DMSO) for the starvation period. As a control, cells were incubated with the FLT3 inhibitor AC220 (20 nM). FLT3 wt expressing THP-1 cells were starved for 4 hours in RPMI medium without FCS, stimulated with FL (200 ng/ml), and harvested sharply 5 min post-stimulation. Stimulation was stopped by transferring the cells to 1.5 ml tubes on ice and subsequently washed once with cold PBS.

Cell pellets were re-suspended in the RIPA lysis buffer (containing 1% Nonidet P-40, 0.25% deoxycholate, 50 mM Tris, pH 7.4, 0.15 M NaCl, 1 mM EDTA freshly supplemented with protease and phosphatase inhibitors) was used. Cells were lysed by vigorous up-and-down pipetting and incubation for 15 min on ice. Then, lysates were cleared by centrifugation (20 min, 13,300 rpm, 4°C). The protein concentration of cleared lysates was measured using a bicinchoninic acid assay (Pierce BCA Protein Assay Kit, Thermo Fisher Scientific) following the manufacturer’s protocol. Samples were prepared in an appropriate sample buffer.

For reliable detection of FLT3 signals, lysates were purified by wheat germ agglutinin (WGA) precipitation. Therefore, 100 – 180 μg of lysate were diluted with immunoprecipitation buffer supplemented with protease and phosphatase inhibitors to a total volume of 700 μl. After adding 30 μl of equilibrated WGA agarose beads (50% slurry, Vector Laboratories, #AL-1023-2), samples were incubated for 3 to 4 hours at 4°C by overhead rotation. Beads were pelleted (3000 rpm, 2 – 3 min, 4°C), and the supernatant was discarded. Beads were washed twice in immunoprecipitation buffer, and precipitated proteins were eluted in 15 μl sample buffer at 95°C for 5 min. To detect other signalling proteins, 15 – 20 μg of lysate were prepared in sample buffer and incubated at 95°C for 5 min. SDS-PAGE and semi-dry blotting were performed as described above.

### SDS-PAGE and immunoblotting

Comparable amounts of proteins were loaded on polyacrylamide (PAA) gels at indicated concentrations. Using standard SDS-PAGE, proteins were separated according to their molecular weight and subsequently blotted to nitrocellulose membrane (Amersham Protran Premium 0.2 μM NC, # 10600001) by tank blotting or semi-dry blotting depending on the size of the proteins of interest. After blotting, membranes were washed, incubated with blocking buffer, and incubated in primary antibody solutions overnight at 4°C. Subsequently, blots were washed, incubated with appropriate horseradish peroxidase–coupled secondary antibodies, and incubated with ECL substrate. The chemiluminescent signal was detected using a LAS-4000 imager (Fujifilm) and quantified using Multi Gauge V3.0 software (Fujifilm). Membranes were first probed with phospho-site specific antibodies and re-probed using the respective α-pan-protein antibody. To re-probe blots, membranes were incubated with Restore PLUS Western Blot Stripping Buffer (Thermo Fisher Scientific, 46430), washed, and re-probed with primary antibody incubation overnight at 4°C.

### PTPRJ phosphatase activity assay

HEK293T cells were seeded in 100 mm dishes and transfected with pBABE-PTPRJ-HA, or empty control plasmids. Cells were harvested using 0.05% Trypsin-EDTA 48 hours post-transfection, washed with cold PBS and lysed with 600 μl PTP lysis buffer (50 mM HEPES pH 7.5; 150 mM NaCl; 5 mM EDTA pH 8.0; 0.5% (v/v) NP40; 1 mM DTT; 5 mM NaF) supplemented with protease inhibitors. Immunoprecipitation of PTPRJ-HA proteins was carried out from 600 to 1000 μg protein lysate with HA antibody (4 – 8 µg) for 2 hours at 4°C by overhead rotating. Immune complexes were immobilized on Protein G-Sepharose beads (Immobilized Protein G, Thermo Fisher Scientific, #. 20399). Beads were washed with lysis buffer (centrifugation 2 – 3 min, 3000 rpm) and incubated with immune complexes for 2 hours at 4°C by overhead rotation. Beads were pelleted (2 – 3 min, 3000 rpm, 4°C), and the supernatant was discarded.

For enzymatic assays, beads were washed once with lysis buffer and twice with PTP reaction buffer (50 mM Sodium Acetate pH 9.0 with freshly added 0.5 mg/ml BSA, 0.5 mM DTT and with or without 5 mM pNPP [Acros Organics; 12886.0100] or 25 µM DiFMUP [Invitrogen; D6567]). Next, samples were incubated in 100 μl complete PTP reaction buffer for 10 – 15 min at 37°C and 750 rpm. A blank (buffer only) was included, and 0.2 mM sodium vanadate was added to one sample to inhibit protein phosphatase activity as a control. After incubation, beads were pelleted, and 50 μl of supernatant were transferred to a 96-well plate (transparent plate for pNPP, and white chimney plate for DiFMUP). The reaction was stopped by adding 50 μl 0.4 N NaOH. Substrate reaction was measured by absorbance at 405 nm for pNPP and fluorescence at 360/465 nm excitation/emission for DiFMUP on a TECAN Infinite F200 plate reader.

To quantify the immunoprecipitated material, samples were subjected to SDS-PAGE and tank blotting. Thus, beads were washed once with PBS, and immune complexes eluted in sample buffer by incubation at 60°C for 10 min. Membranes were immunologically probed against PTPRJ or HA.

### Assessing TMD-TMD interaction with the DN-AraTM transcriptional reporter assay

The DNA sequences coding for the TMD of FLT3 and PTPRJ were cloned into either pAraTMwt (coding for AraC) or pAraTMDN (coding for the inactive form of AraC, AraC*) plasmids, as previously described (38). Unless otherwise stated, standard molecular biology techniques were used, and all constructs were verified by DNA sequencing (Genewiz, Inc.). The protein sequences for both FLT3 and PTPRJ used in AraTM assays contains five extracellular residues, the putative TMD as predicted by MPEx (underlined below) (68), and 20 cytoplasmic juxtamembrane residues:

FLT3 - QDNISFYATIGVCLLFIVVLTLLICHKYKKQFRYESQLQMVQVTG
PTPRJ - QDPGVICGAVFGCIFGALVIVTVGGFIFWRKKRKDAKNNEVSFSQIKP

The constructs and the reporter plasmid (pAraGFPCDF) were co-transformed into the AraC-deficient *E. coli* strain SB1676 and streaked onto selective plates. Colonies were picked from each construct and grown in LB media for 8 h at 30 °C. Each culture was diluted into selective autoinduction media and grown for an additional 16 h at 30 °C. A series of 2-fold dilutions of the cultures were prepared in a black 96-well, clear-bottom plate. Absorption at 580 (10) nm and GFP fluorescence emission spectra (excitation maximum 485 (20) nm and emission maximum at 530 (30) nm were collected using an Infinite^®^ 200 PRO Plate Reader (Tecan). The results are reported as the ratio of fluorescence emission at 530 nm to absorbance at 580 nm and normalized to the negative control (empty plasmids and reporter plasmid). Immunoblotting was performed using HRP-conjugated anti-maltose binding protein (MBP) monoclonal antibody at 1:10000 dilution (New England Biolabs, #E8038) to verify expression levels of each construct.

### Analysis of cell proliferation and viability

For MV4-11 cells, proliferation and viability were analyzed by trypan blue count and flow cytometry using cell counting beads. For viability assessment, 2x 10 μl per sample was taken, and the percentage of dead cells was measured by automated trypan blue count (Countess II FL, Invitrogen). For flow cytometric cell count, samples were mixed with 10 μl 123count eBeads (Invitrogen, catalog no. 01-1234-42) and measured on a BD FACS Canto.

The transformative capacity of cells was analyzed by colony-forming unit (CFU) assay. Cells were grown in a 1.27% methylcellulose-containing, semi-solid medium. Cells were seeded in triplicates at a density of 500 cells/well (MV4-11, THP-1) or 400 cells/well (32D FLT3 ITD) in 24-well plates. The remaining wells were filled with PBS, and plates were placed in humidity chambers for cultivation. After 7 to 10 days, colonies were stained by adding 50 μl of iodonitrotetrazolium chloride solution (4 mg/ml) dropwise to the wells. After overnight incubation, plates were scanned on an HP Scanjet G4050 photo scanner, and the number of colonies was counted using ImageJ v1.53k software (National Institutes of Health).

## Results

The TMD residues G979, G983, and G987 of PTPRJ have been previously identified as major mediators of PTPRJ self-association (38) (54). Glycine-to-leucine (G-to-L) mutations of those residues destabilize PTPRJ oligomerization and increase phosphatase activity towards EGFR by promoting substrate access (38). We hypothesized that TMD mutants of PTPRJ would have similar effects on FLT3 phosphorylation and signalling.

To assess if an altered activity of PTPRJ TMD mutants would interfere with cellular signal transduction processes, model cell systems expressing the FLT3 wt (THP-1), constitutive active oncogenic FTL3 ITD (MV4-11), or ectopic FLT3 ITD (32D) were used. Leukemic human THP-1 cells allow to study the effects of PTPRJ on the FLT3 wt receptor. Both, the human leukemic cell lines MV4-11 expressing biallelic FLT3 ITD as well as murine 32D, stably transduced with FLT3 ITD are factor independent cells lines. They rely on the constitutive kinase activity of mutant FLT3 ITD and are routinely used by us and others to study effects on modified FLT3 ITD kinase activity (22, 64, 66, 69–72). Since PTPRJ is a well-characterized antagonistic phosphatase for FLT3 proteins, these cell systems provide clear input about the *in situ* activity of mutant PTPRJ. To prevent interference from endogenous wt PTPRJ, we inactivated chromosomally encoded PTPRJ in these cells using CRISPR/Cas9. Biallelic gene inactivation was confirmed by DNA sequencing, and the absence of PTPRJ production was confirmed by immunoblotting (data not shown). Subsequently the cell lines were transduced with retroviral particles encoding PTPRJ wild type or G-to-L TMD mutant PTPRJ variants. In order to compare the effect of altered oligomerization on FLT3 activity and downstream signaling, cell lines were sorted for similar phosphatase expression by flow cytometric cell sorting using CD148-APC-mediated PTPRJ surface labeling. Unfortunately, stable cell populations with similar PTPRJ protein levels could not be obtained in all cell populations modified. Thus, only cell lines with similar PTPRJ protein levels were used in the study (**Supplementary Figure 1**).

### PTPRJ TMD mutants decrease FLT3 phosphorylation

**Figure 1:**
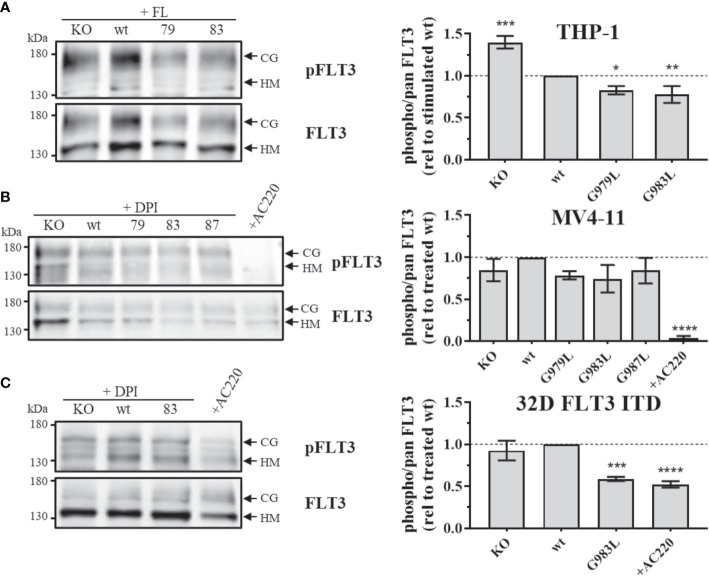
Phosphorylation of FLT3 in MV4-11, THP-1, and 32D FLT3 ITD PTPRJ KO AML cell lines stably re-expressing PTPRJ wt or TMD G979L, G983L, or G987L mutants. Cells were starved for 4 h in serum-free medium and lysed in RIPA buffer. During starvation, MV4-11 and 32D FLT3 ITD cells were treated with DPI (0.5 µM) or AC220 (20 nM), as indicated. THP-1 cells were stimulated with FL (200 ng/ml, 5 min) before harvest. FLT3 was enriched from 100 – 180 µg lysate by wheat germ agglutinin precipitation and subjected to SDS-PAGE and immunoblotting. Blots were first probed by phospho-site specific antibodies recognizing FLT3 pY591 and re-probed for total FLT3. **(A–C)** *Left:* Representative immunoblots (n = 3) are shown. Positions of immature, high mannose (HM, 130 kDa) and mature, complex glycosylated (CG, 160 kDa) forms of FLT3 are indicated by arrows. Positions of 130 and 180 kDa molecular weight standard bands are shown on the left side of the blots. 79 – G979L; 83 – G983L; 87 – G987L. *Right*: Quantification of specific FLT3 phosphorylation. Values were calculated as the ratio of phosphorylated to total receptor (sum of HM and CG signal) and normalized to DPI-treated or FL-stimulated wt. All values are given as mean ± standard deviation, n = 3. Statistics: one-way ANOVA, followed by Dunnett’s multiple comparisons tests; *p ≤ 0.05; **p ≤ 0.01; ***p ≤ 0.001; ****p ≤ 0.0001 compared to wt.

If PTPRJ TMD mutants show enhanced phosphatase activity *in situ*, diminished FLT3 phosphorylation and downstream signalling would be expected. In THP-1 cells, expression of PTPRJ G979L or G983L resulted in significantly reduced FL-mediated receptor phosphorylation at position Y591 in comparison to wt PTPRJ, indicating decreased FLT3 activity in those cells (**Figure 1A**) (39). Because FLT3 ITD induces high levels of cellular ROS with subsequent oxidative inactivation of PTPRJ (Godfrey 2012, Jayavelu 2016), cell lines expressing FLT3 ITD (MV4-11 and 32D) were treated with DPI during starvation to quench ROS production. While a weak reduction of Y591 phosphorylation could be observed in response to the expression of PTPRJ TMD mutants in MV4-11 cells (**Figure 1B**), the expression of G983L PTPRJ in 32D FLT3 ITD cells resulted in significantly reduced FLT3 phosphorylation (**Figure 1C**).

### Mutations in the TMD of PTPRJ affect receptor oligomerization states but not PTP activity

**Figure 2:**
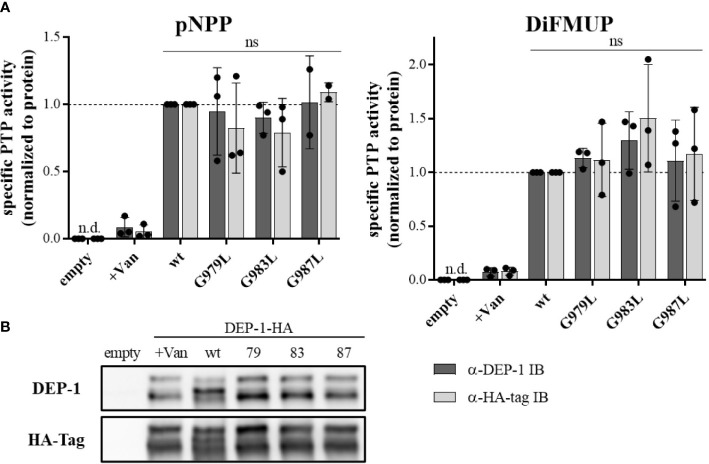
Specific PTP activity of wt and TMD mutant PTPRJ remains unchanged PTPRJ-HA proteins were immunoprecipitated from transiently transfected HEK293T cells with pBABE-PTPRJ-HA constructs (wt, G979L, G983L, and G987L) 48 hours post-transfection. Cell lysates were probed with phosphatase substrate mimetics pNPP or DiFMUP for 10 – 15 min at 37°C. Vanadate was added to controls (0.2 mM, +Van) to inhibit phosphatase activity. Substrate cleavage was measured by absorbance at 405 nm (pNPP) or fluorescence at 360/465 nm ex/em (DiFMUP). The amount of immunoprecipitated protein was analyzed by immunoblotting using antibodies recognizing PTPRJ and HA-tag. **(A)** Specific PTP activity of PTPRJ variants relative to respective wt is shown. Values were calculated as absorbance, or fluorescence measurements minus blank (buffer only) normalized to the amount of immunoprecipitated protein, based on quantification of PTPRJ or HA-tag immunoblots (IB). The individual values of three independent experiments (for pNPP G987L n = 2) are shown by closed circles, columns, and error bars represent mean ± standard deviation. Statistics: two-way ANOVA and Dunnett’s multiple comparisons test; (ns, not significant; n.d., not detectable). **(B)** A representative immunoblot is shown (n = 3). 79 – G979L; 83 – G983L; 87 – G987L.

To address the question, of whether altered PTP oligomerization is due to altered substrate accessibility or due to altered specific enzymatic activity, PTPRJ variants were immunoprecipitated and specific phosphatase activity was determined. In comparison to wt PTPRJ immunoprecipitated HA-tagged PTPRJ TMD mutants showed no significant changes in activity towards pNPP and DiFMUP (**Figure 2**). Thus, it can be concluded that the observed diminished FLT3 dephosphorylation is not due to an altered specific PTP activity of the PTPRJ TMD mutants but most likely to changes in PTPRJ.

**Figure 3:**
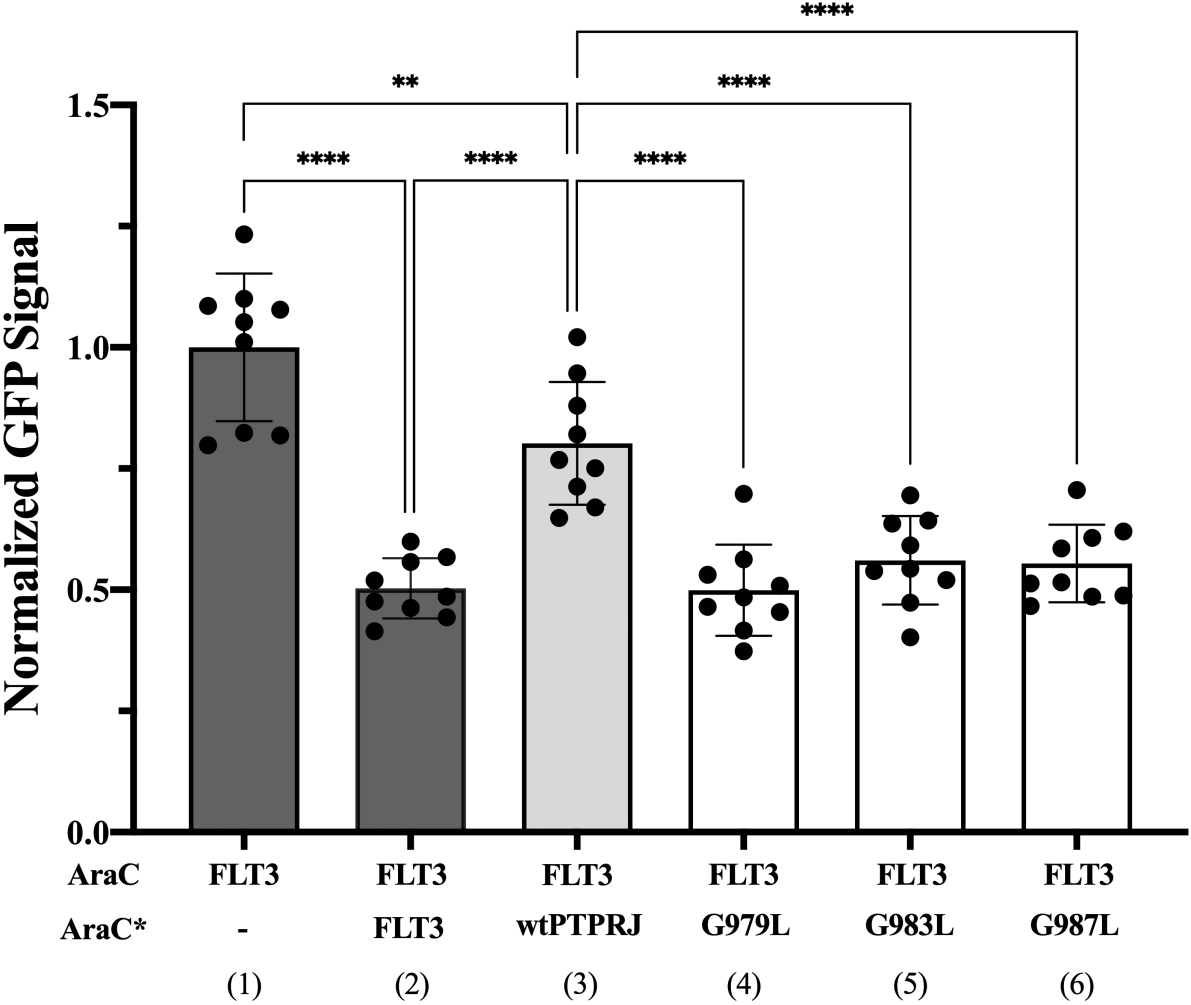
Quantifying FLT3 self-association and hetero-interaction with wt PTPRJ and 3 TMD point mutants using the DN-AraTM assay. GFP signal is normalized to the signal of FLT3 (1), and the results are shown as mean ± S.E. (n=9). Pair-wise statistical significance (1 vs. 3 and 2 vs. 3) was assessed using an unpaired t-test (at 95% confidence intervals). Results were normalized, taking into account the slight differences in expression level measured by immunoblotting (**Supplementary Figure 2**). Statistical significance was assessed using one-way ANOVA followed by Dunnett’s multiple comparisons test at 95% confidence intervals. ****p ≤ 0.0001; **p ≤ 0.01.

We then sought to assess whether FLT3 and PTPRJ may interact through direct TM interaction and whether introducing the glycine-to-leucine mutations not only disrupts PTPRJ self-association but also promotes interaction between the TMDs of PTPRJ and FLT3. To do so, we used the dominant-negative AraC-based transcriptional reporter assay (DN-AraTM), an assay reporting on the propensity of TMD to self-associate and heterodimerize in cell membranes (38, 73–77). When the TMD of FLT3 was expressed as a fusion to AraC, a strong GFP signal was observed, indicating that the TMD has a high propensity to self-associate (**Figure 3**; lane 1). On the other hand, when FLT3-AraC* was co-expressed as a competitor to FLT3-AraC, a significant decrease in GFP signal was observed (**Figure 3**; lane 2), consistent with specific TMD-TMD interactions. The results show that while wt PTPRJ has a propensity to associate with FLT3 (**Figure 3**; lane 3), it does so to a lesser extent than FLT3 itself (**Figure 3**; lane 2 vs. lane 3). Importantly, the PTPRJ mutants show a significant decrease in GFP signal compared to wt PTPRJ, consistent with a more productive association with FLT3 (**Figure 3**; lane 3 vs. lanes 4-6).

Altogether, these results suggest that PTPRJ’s self-association prevents its access to FLT3 and that disrupting it through TMD mutations promotes interaction with FLT3 and subsequent receptor dephosphorylation.

### PTPRJ TMD mutants decrease FLT3 downstream signalling

To further investigate the effect of PTPRJ mutations on FLT3 activity, phosphorylation of signalling proteins downstream of FLT3 was analyzed. Whereas wt FLT3 signals mainly through PI3K/AKT and RAF/MAPK cascades upon ligand stimulation, constitutively active FLT3 ITD predominantly induces STAT5 signalling due to altered localization in the ER (8, 9, 16, 19).

**Figure 4:**
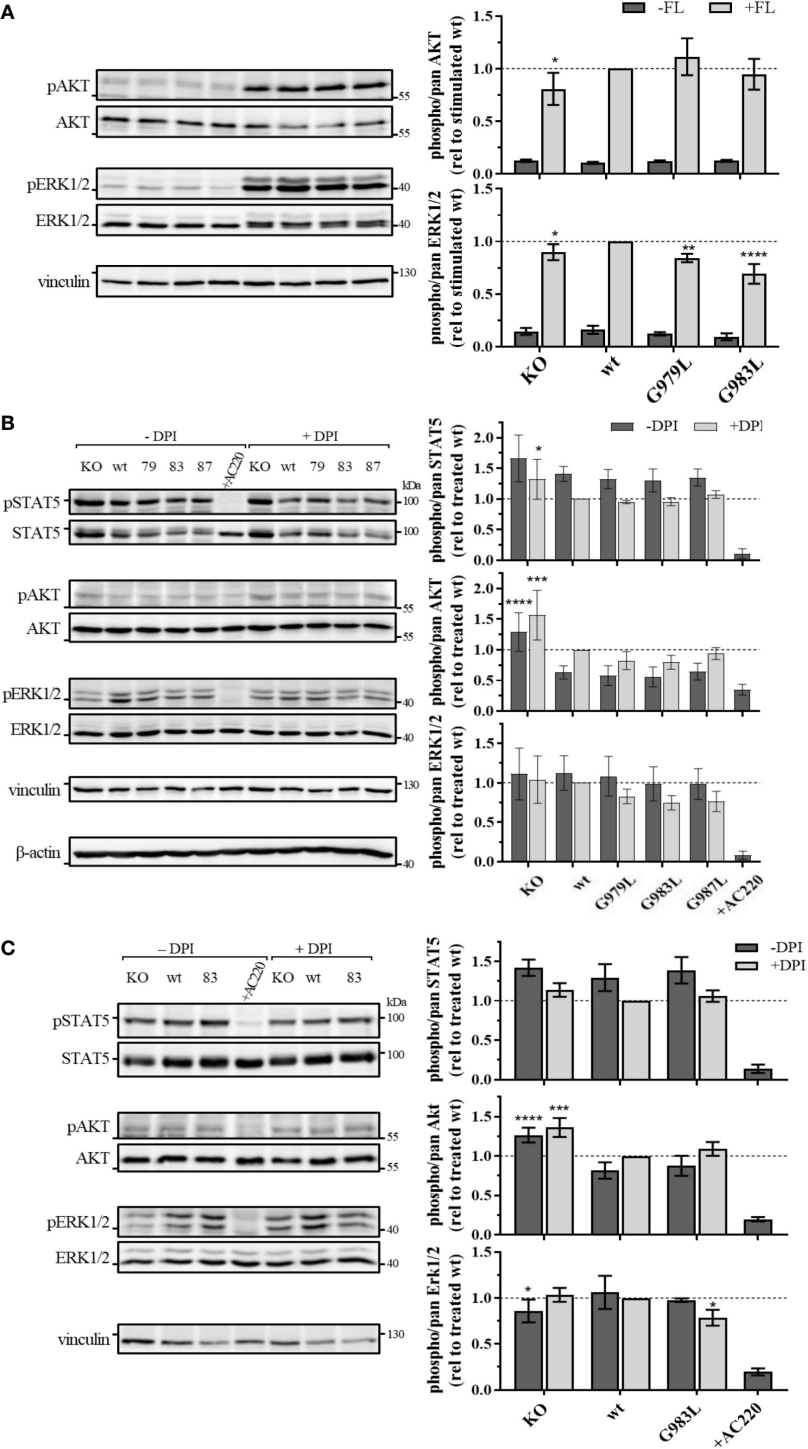
Signaling analysis of THP-1 **(A)** MV4-11 **(B)** and 32D FLT3 ITD cells expressing TMD mutant PTPRJ. **(A)** THP-1 PTPRJ KO cells or cells re-expressing PTPRJ wt, G979L, or G983L were starved for 4 h in serum-free medium, then stimulated with 200 ng/ml FLT3 ligand for 5 min (+FL) or left unstimulated (–FL) and lysed in RIPA buffer. **(B, C)** MV4-11 and 32D FLT3 ITD PTPRJ KO cells or cells re-expressing PTPRJ wt, G979L, G983L, or G987L were starved for 4 h in serum-free medium and treated with DPI (0.5 µM), AC220 (20 nM), or mock (DMSO), as indicated, then lysed in RIPA buffer. Equivalent amounts of protein samples were separated by SDS-PAGE and blotted to a nitrocellulose membrane. Blots were first probed by phospho-site specific antibodies recognizing pSTAT5 (Y694), pAKT (S473), and pERK1/2 (T202/Y204). Blots were re-probed for total STAT5, AKT, and ERK1/2 and subsequently analyzed with antibodies recognizing vinculin (124 kDa) and beta-actin (42 kDa) as a loading control. *Left*: Representative immunoblots are shown. Dashes indicate positions of molecular weight standard bands. 79 – G979L; 83 – G983L; 87 – G987L *Right*: Quantification of specific phosphorylation of AKT, ERK1/2, and STAT5 in relation to the total protein level. Values were normalized to FL-stimulated or DPI treated wt and are given as mean ± standard deviation, n = 3 - 4. Statistics: two-way ANOVA and Dunnett’s multiple comparisons test; *p ≤ 0.05; **p ≤ 0.01; ***p ≤ 0.001; ****p ≤ 0.0001 compared to respective wt.

In THP-1 cells, strong activation of AKT and ERK1/2 could be observed upon FL stimulation (**Figure 4A**), illustrating the main signalling pathways of wt FLT3. No phosphorylation of STAT5 at position Y694 was detected (data not shown). FL-induced phosphorylation of ERK1/2 MAPK kinases was significantly decreased in cells expressing PTPRJ G979L and G983L compared to wt PTPRJ. But no alteration in AKT S473 phosphorylation could be observed. In MV4-11 cells, endogenously expressed FLT3 ITD induces STAT5 activation, as indicated by prominent Y694 phosphorylation, and activation through ERK1/2 and AKT, albeit less pronounced (**Figure 4B**). FLT3 ITD mediated activation of these pathways was confirmed by the substantial reduction of STAT5, AKT, and ERK1/2 phosphorylation upon treatment with the FLT3 inhibitor AC220. Treatment with DPI to prevent the ROS-mediated inhibition of PTPRJ resulted in reduced STAT5 and ERK1/2 phosphorylation, confirming the restored PTPRJ phosphatase activity. Overexpression of FLT3 ITD in 32D cells resulted in strong constitutive activation of STAT5 as well as RAF/MAPK and, more weakly, PI3K/AKT signalling (**Figure 4C**). Abrogation of activation upon AC220 treatment demonstrated FLT3 ITD mediated induction. Similarly to MV4-11 cells, DPI treatment showed diminished STAT5 phosphorylation, indicating PTPRJ activation.

Despite the significant reduction in FLT3 Y591 phosphorylation upon expression of the PTPRJ TMD mutants (**Figure 1**), reduction of FLT3 ITD downstream signalling could only be observed in 32D FLT3 ITD cells expressing PTPRJ G983L, in which a decrease in ERK1/2 phosphorylation is observed in response to DPI treatment (**Figure 4C**). Taken together, diminished ERK1/2 phosphorylation in wt and FLT3 ITD cell lines expressing PTPRJ TMD mutants further indicates activation of PTPRJ upon abrogation of oligomerization.

### PTPRJ TMD mutants decrease FLT3-driven cell proliferation and clonal growth

**Figure 5:**
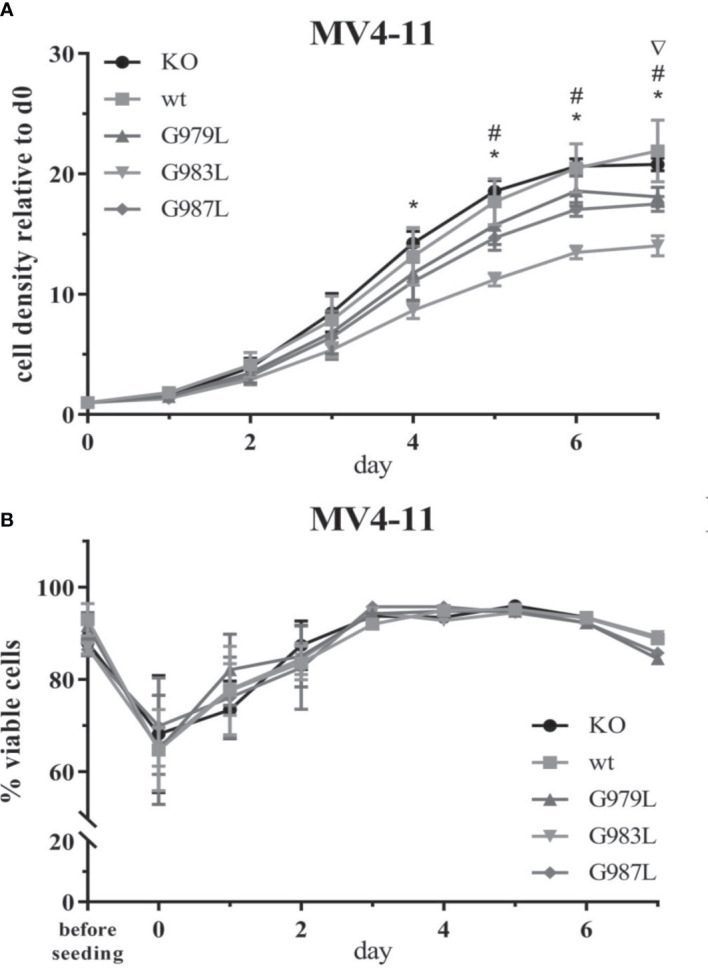
Proliferation and viability of MV4-11 cells expressing TMD mutant PTPRJ proteins. A+B) MV4-11 PTPRJ KO cells re-expressing wt PTPRJ or G-to-L mutants were seeded in duplicates in a 6-well plate. Cells were counted 4 hours after seeding (= day 0) and then every 24 hours for 7 days. **(A)** Cell density was determined by flow cytometry by measuring the number of viable cells in relation to the number of counting beads. The graphs show cell density relative to day 0. Values are given as mean ± standard deviation, n = 3. Statistics: repeated-measures two-way ANOVA and Dunnett’s multiple comparisons test; significant differences (p ≤ 0.05) of TMD mutants G979L (∇); G983L (*); and G987L (#) to wt PTPRJ are indicated. **(B)** Percentage of viable cells, measured by automated trypan blue count, is shown. Values are given as mean ± standard deviation, with n = 3 for d0 – 2 and n = 1 for d3 – 7.

To study the phenotypic consequences of the enhanced activity of PTPRJ TMD mutants towards FLT3, proliferation, viability, and transformation capacity of MV4-11 cells were assessed. The overexpression of wtPTPRJ did not result in an impaired proliferation of MV4-11 cells compared to cell with inactivated endogenous PTPRJ. In contrast, proliferation was significantly decreased in the MV4-11 cell lines expressing PTPRJ TMD mutants (albeit to a different extent) when compared to wt PTPRJ (**Figure 5A**). The G983L mutation, which has been previously shown to be the most impactful in disrupting PTPRJ oligomerization and in inhibiting EGFR-driven phenotypes (38), exhibited the most substantial effect on the proliferative capacity of all mutants. On the other hand, the expression of the TMD mutants did not significantly impact cell viability. (**Figure 5B**).

**Figure 6:**
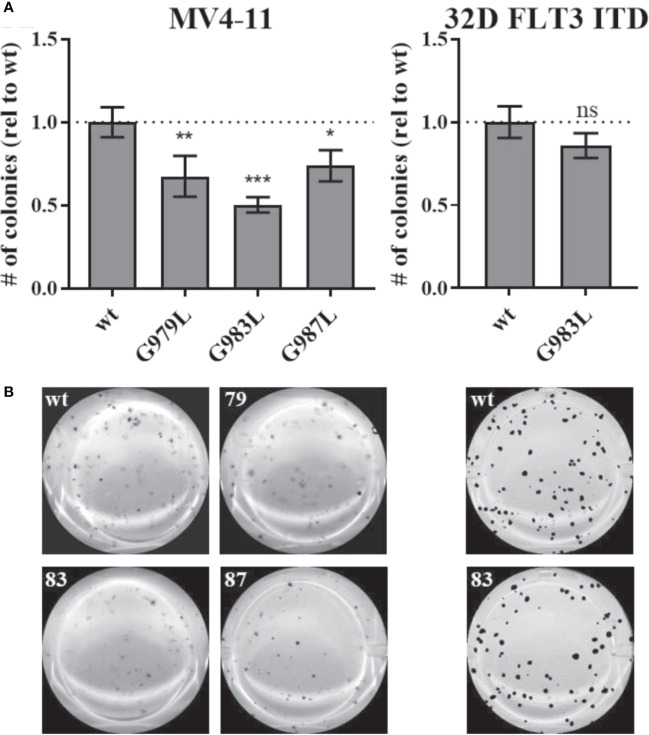
Clonal growth of FLT ITD cells expressing TMD mutant PTPRJ proteins. MV4-11 and 32D FLT3 ITD PTPRJ KO cells re-expressing PTPRJ wt, G979L, G983L, or G987L were seeded in triplicates in 1.27% methylcellulose-containing, cytokine-free medium. Colonies were stained with iodonitrotetrazolium chloride after 7 days (32D FLT3 ITD) or 10 days incubation (MV4-11) and quantified using Image (J) **(A)** Number (#) of colonies per well relative (rel) to wt is shown. Values are given as mean ± standard deviation, n = 3. Statistics for MV4-11: one-way ANOVA and Dunnett’s multiple comparisons test; *p ≤ 0.05; **p ≤ 0.01; ***p ≤ 0.001 compared to respective wt (ns; not significant). Statistics for 32D FLT3 ITD: unpaired two-tailed t-test; * p ≤ 0.05. **(B)** Representative images of whole wells are shown. 79 – G979L; 83 – G983L; 87 – G987L.

Constitutive activity of FLT3 ITD drives cellular transformation *via* AKT, STAT5, and RAS pathways (10, 78). PTPRJ depletion and ROS-induced inhibition have been previously shown to promote leukemic cell transformation due to elevated activity of FLT3 and downstream signalling (22, 24, 39). We hypothesized that disrupting PTPRJ oligomerization and thereby presumably enhancing its activity could impair FLT3-driven transformation of AML cells. Therefore, *in vitro, the* transformative capacity of cell lines stably expressing PTPRJ TMD mutants was analyzed compared to wt PTPRJ cells. Cells were grown in semisolid, cytokine-free methylcellulose medium, and colony formation was quantified after 7-10 days. MV4-11 cells exhibited a significantly reduced clonal growth in methylcellulose for all three PTPRJ TMD mutants compared to wt (**Figure 6**), indicating restriction of FLT3 ITD activity. The strongest impairment of transformation capacity was observed in PTPRJ G983L–expressing cells. 32D FLT3 ITD cell lines demonstrated IL3-independent, FLT3 ITD–driven clonal growth, as previously described (10, 72). Colony formation was only insignificantly reduced in PTPRJ G983L–expressing cells compared to wt.

### PTPRJ TMD mutations induce alterations in global protein tyrosine phosphorylation

Next, we addressed how altered PTPRJ self-association influenced global protein tyrosine phosphorylation in cells expressing either wt FLT3 or FLT3 ITD. Possible changes could be attributed directly to enhanced PTPRJ phosphatase activity and inhibition of FLT3 kinase activity.

First, global protein phosphorylation was analyzed by immunoblotting using pY-site-specific antibodies. To abrogate FLT3-independent signalling events, cells were starved for 4 hours in serum- and cytokine-free medium. MV4-11 and 32D cells with constitutively active FLT3 ITD were then harvested directly, whereas wt FLT3 in THP-1 cells was first stimulated with FL (200 ng/ml, 5 min). Cells were then lysed, and lysates were analyzed by SDS-PAGE and immunoblotting. In THP-1 cells, global pY increased by more than 2-fold upon FL stimulation, illustrating phosphorylation events downstream of activated wt FLT3 (**Supplementary Figure 3A**). Upon ligand stimulation, protein tyrosine phosphorylation of cells stably expressing PTPRJ G983L was significantly reduced compared to PTPRJ wt cells.

To quench cellular ROS levels of FLT3 ITD–expressing cells, DPI was added during starvation before analysis of protein phosphorylation. Here, no consistent effect of DPI treatment on MV4-11 cells regarding global protein tyrosine phosphorylation was observed (**Supplementary Figure 3B**). In 32D FLT3 ITD cells expressing PTPRJ wt or G983L, pY was slightly decreased upon DPI treatment, suggesting restored PTPRJ phosphatase activity (**Supplementary Figure 3C**).

In DPI-treated and untreated MV4-11 cells, the global protein tyrosine phosphorylation was found to be slightly reduced in PTPRJ G983L cells compared to wt, but without a statistically significant difference (**Supplementary Figure 3B**). The clear reduction in pY upon treatment with the FLT3 inhibitor AC220 demonstrates the dependency of observed phosphorylation events on FLT3 ITD activity. In contrast, in 32D FLT3 ITD cells, no differences in global pY of cells expressing PTPRJ wt and TMD were observed, irrespective of DPI treatment (**Supplementary Figure 3C**). As expected, 32D FLT3 ITD PTPRJ KO cells showed an increased global pY compared to 32D FLT3 ITD cells re-expressing PTPRJ. Surprisingly, AC220 treatment only slightly reduced protein phosphorylation in 32D FLT3 ITD cells, suggesting fewer FLT3 ITD–dependent phosphorylation events or incomplete inhibition of FLT3 activity.

Taken together, these findings with MV4-11 cells indicate increased dephosphorylation of PTPRJ targets and impaired FLT3 signalling in the presence of PTPRJ TMD mutants, especially G983L.

## Discussion

PTPRJ has been shown to act as an antagonistic regulator on FLT3 activity *in vitro* and *in vivo (* 39, 79 *).* and its oligomerization has been demonstrated to hinder its activity (38, 58). Therefore, disruption of its PTPRJ oligomerization could result in an *in situ* activation of its activity and would consequently result in decreased FLT3 receptor phosphorylation. Data presented here elucidated the effects on FLT3 signalling of TMD mutations known to disrupt PTPRJ oligomerization. First, determination of the specific phosphatase activity of immunoprecipitated PTPRJ revealed no alteration of phosphatase activity of mutant PTPRJ. Using our established leukemic model cell lines inactivated for endogenous PTPRJ but stably re-expressing TMD mutated PTPRJ variants phosphorylation of FLT3/FLT3 ITD and downstream targets, as well as cellular proliferation, viability, and clonal growth, were studied. Diminished phosphorylation of wt FLT3 and FLT3 ITD in AML cell lines re-expressing PTPRJ TMD mutants compared to cells expressing wt PTPRJ was accompanied by reduced downstream signalling *via* ERK1/2 and selective PTPRJ substrates as shown by mass spectrometry. Lastly, PTPRJ TMD mutant proteins impaired proliferation and *in vitro* transformation of leukemic MV4-11 cells. These findings reveal an enhanced activity of PTPRJ TMD mutant proteins *in situ*. Of the three studied PTPRJ G-to-L substitutions, G983L (a TMD mutation shown to be the most disruptive of PTPRJ oligomerization) seemed to have the most potent effects on FLT3 activity and tyrosine phosphorylation as well as cellular proliferation and clonal growth.

Wild type FLT3 and FLT3 ITD have been established as *bona fide* substrates of PTPRJ (24, 39, 40). Accordingly, PTPRJ depletion in wt FLT3-expressing 32D and THP-1 cell lines has been shown to increase FL-stimulated FLT3 phosphorylation (e.g., at Y589, Y591, or Y842). In contrast, PTPRJ–overexpressing cells were characterized by decreased receptor phosphorylation (39). Similarly, co-expression of PTPRJ and FLT3 ITD in HEK293T cells diminished FLT3 ITD phosphorylation in ROS-quenched cells (39). As expected, our immunoblotting results revealed significantly decreased phosphorylation of wt FLT3 and FLT3 ITD at the PTPRJ regulated Y591 site (39) in leukemic cells expressing PTPRJ TMD mutants. Phosphorylation of this residue is considered a hallmark of FLT3 kinase activation and is presumed to be involved in relieving the autoinhibitory conformation of the FLT3 JM domain (4, 5). Y591 becomes phosphorylated by autocatalysis upon ligand stimulation and is constitutively phosphorylated in FLT3 ITD (16, 80, 81).

Chemical *in situ* crosslinking was used to reveal the effect of TMD G-to-L replacements on their oligomerization at the membrane. It should be noted that immunologically detected high molecular weight of crosslinked PTPRJ-HA could represent PTPRJ homodimers but could include other proteins. Analyses by Iuliano et al. using a mostly identical BS3-crosslinking protocol identified the crosslinked PTPRJ species as homodimers (82). However, another study based on PTPRJ TMD protein chimeras with staphylococcal nuclease observed higher order oligomers in SDS micelles in addition to dimers (54). By analysing specific PTP activity of immunoprecipitated PTPRJ, no distinct effects of PTPRJ TMD mutations could be detected. Moreover, our results show that the TMDs of FLT3 and PTPRJ have a propensity to interact with each other and that the PTPRJ TMD mutants enhance this interaction. Together, our findings indicate that the PTPRJ G-to-L mutations promote the dephosphorylation of FLTR3 (and thus reducing its activity) not by affecting PTPRJ’s specific phosphatase activity but by destabilizing its self-association and favouring its access to FLT3 in the cellular membrane as a monomer. Moreover, our results suggest that decreased FLT3 activity might also result from a disruption of FLT3 dimer by PTPRJ TMD mutants (**Figure 3** **; lane 1 vs. lanes 4-6**).

While PTPRJ is presumed to be primarily localized at the plasma membrane (37, 46, 83), FLT3 ITD is retained in its immature HM form in the ER/Golgi system (17, 66). The maturation efficiency of FLT3 ITD has been demonstrated to be inversely correlated to its tyrosine phosphorylation (17, 66). PTP such as SHP1 and PTP1B have been found to dephosphorylate FLT3 ITD (66). In agreement with those findings, increased activity of the PTPRJ G983L mutant on FLT3 tended to enhance FLT3 ITD maturation. In addition, maturation seemed to be slightly decreased in *PTPRJ* KO cells, consistent with observations in bone marrow cells isolated from *PTPRJ* KO FLT3 ITD knock-in mice (84).

While treatment with the FLT3 inhibitor AC220 completely abrogated FLT3 ITD auto-phosphorylation in MV4-11 cells, it surprisingly reduced receptor phosphorylation in 32D FLT3 ITD cells only by ca. 50%. However, downstream signalling of FLT3 ITD in AC220-treated 32D cells was diminished, demonstrating that FLT3 activity was efficiently suppressed despite the residual pY591 signal. Those findings are consistent with a previous study using identical treatment conditions and cell lines (70). In a similar FLT3 overexpression system in 32D cells, Kellner and colleagues characterized FLT3 ITD to have a slower protein turnover than the wt receptor (71). This increased half-life of FLT3 ITD could explain why phosphorylated protein was still detectable in AC220-treated cells.

Like receptor phosphorylation, ERK1/2 phosphorylation downstream of wt FLT3 has been previously described to be enhanced in PTPRJ–depleted THP-1 or 32D cells (39). Likewise, signalling *via* STAT5 increases upon PTPRJ knockdown in DPI-treated 32D FLT3 ITD cells (24). As expected, ERK1/2 phosphorylation at T202/Y204 was impaired in both wt FLT3 and FLT3 ITD harbouring cell lines upon expression of PTPRJ TMD mutants. In addition to reduced activation downstream of inhibited FLT3, this could be partly due to the enhanced action of PTPRJ on ERK1/2 Y204, which has been previously identified as a direct target of PTPRJ phosphatase activity (85). In contrast, AKT activation was mostly unchanged by the expression of PTPRJ TMD mutant proteins.

Physiological alterations in leukemic cell lines due to the enhanced activity of PTPRJ TMD mutants were demonstrated. In particular, PTPRJ has been previously shown to play a role in suppressing FLT3-mediated leukemic cell transformation (22, 24, 39). Reactivating PTPRJ by counteracting ROS has been found to attenuate the clonal growth of 32D FLT3 ITD cells (22, 24). Furthermore, shRNA-mediated depletion of PTPRJ has been shown to induce FL-stimulated colony formation of 32D cells stably expressing wt FLT3 (39). Here, FLT3 ITD–driven clonal growth was reduced for MV4-11 cells stably expressing PTPRJ TMD mutants, and the same trend was observed with 32D FLT3 ITD cells. Similarly, the reactivation of PTPRJ’s PTP activity by blocking ROS has been demonstrated to prolong survival in a murine model of FLT3 ITD–driven myeloproliferative disease (22, 24).

Expression of PTPRJ TMD mutants in MV4-11 cells significantly impaired their proliferation, again indicating enhanced activity of TMD mutant PTPRJ against FLT3 ITD and/or other targets. In contrast, expression of PTPRJ G983L did not affect the proliferation of 32D FLT3 ITD cells within the four days of observation. This could result from high ROS levels and subsequent oxidative inactivation of PTPRJ. It also seems possible that prolonged growth of cells might be required due to the strong stimulatory effect of overexpressed FLT3 ITD on proliferation. Similarly, the impact of PTPRJ depletion on FL-induced proliferation of 32D FLT3wt cells has been previously found to be only moderate when scored after three days but much more pronounced after five days (39).

Total pY immunoblotting provided insight into the effect of PTPRJ on global Tyr phosphorylation. Immunoblotting revealed global pY to be significantly reduced in THP-1 cells expressing PTPRJ G983L compared to PTPRJ wt. The same trend was observed in MV4-11 cells. Thus, our data indicate enhanced PTP activity of TMD mutant PTPRJ and/or reduced FLT3 activity in those cells. Taken together, our findings provide clear evidence that disrupting PTPRJ TMD–mediated self-association may be used as a tool to restrict oncogenic FLT3 activity. Therefore, the potential application of PTPRJ TMD-targeting peptides (38) in treating FLT3 ITD–positive AML should be investigated further.

## Data availability statement

The original contributions presented in the study are included in the article/**Supplementary Material**. Further inquiries can be directed to the corresponding author.

## Author contributions

JPM and DT conceived the project; MS, SR, WE, and AK performed the experiments. MS, DT, and JPM wrote the manuscript. All authors contributed to the article and approved the submitted version.

## Funding

This work was supported by the Deutsche Forschungsgemeinschaft, grant Mu955/14-1 and Mu955/15-1 to JM, and by the National Institute of General Medical Sciences [grant number R01GM139998] to DT.

## Conflict of interest

The authors declare that the research was conducted in the absence of any commercial or financial relationships that could be construed as a potential conflict of interest.

## Publisher’s note

All claims expressed in this article are solely those of the authors and do not necessarily represent those of their affiliated organizations, or those of the publisher, the editors and the reviewers. Any product that may be evaluated in this article, or claim that may be made by its manufacturer, is not guaranteed or endorsed by the publisher.

## Supplementary material

Supplementary Figure 1 Stable re-expression of PTPRJ in AML cell lines. PTPRJ wt and G-to-L variants were stably expressed in MV4-11, THP-1, and 32D FLT3 ITD PTPRJ KO cells by retroviral transduction. Cells were sorted for similar PTPRJ surface expression levels using flow cytometric based on PTPRJ detection. **(A)** PTPRJ surface expression. Cells were stained with APC-coupled α-CD148 (PTPRJ) antibody, and the APC signal was measured flow cytometrically. Histograms show cell-bound APC fluorescence intensity (APC-A). **(B)** Cellular PTPRJ expression level. Cell lysates were separated by SDS-PAGE, blotted, and analyzed with antibodies recognizing PTPRJ. For loading control, antibodies recognizing vinculin (124 kDa) and beta-actin (42 kDa) or Ponceau staining (not shown) were used. Graphs show PTPRJ signal normalized to loading control (ctrl), relative (rel) to respective wt. Values are given as mean ± standard deviation, with n = 5 – 6 for MV4-11 and n = 3 for THP-1 and 32D, except for MV4-11 and THP-1 parental n = 1. Statistics: one-way ANOVA and Dunnett’s multiple comparisons test; * p ≤ 0.05 compared to respective wt. ns – not significant. Representative immunoblots are shown below graphs. Positions of 40 and 130 kDa bands of molecular weight standard are indicated. 79 – G979L; 83 – G983L; 87 – G987L.

Supplementary Figure 2 Representative immunoblot analysis against MBP used to normalize the DN-AraTM assay results. Bacteria are always transformed with both AraC* and AraC-containing plasmids. The dash (-) represents an empty vector without the TMD construct of interest.

Supplementary Figure 3 Global protein tyrosine phosphorylation pattern of DEP-1 wt and TMD mutant expressing cell lines. THP-1 **(A)**, MV4-11 **(B)**, and 32D FLT3 ITD **(C)** PTPRJ KO cells or cells re-expressing DEP-1 wt, G979L, G983L, and G987L were starved for 4 h in serum- and cytokine-free medium and lysed in RIPA buffer. During starvation, MV4-11 and 32D FLT3 ITD cells were treated with DPI (0.5 µM), AC220 (+AC, 20 nM), or mock (DMSO), as indicated. THP-1 cells were stimulated with FL (200 ng/ml, 5 min) before harvest. Equivalent amounts of protein samples (15 – 20 µg) were separated by SDS-PAGE, blotted to a nitrocellulose membrane, and analyzed for global tyrosine phosphorylation using α-pY antibody (4G10). Blots were re-probed for vinculin (124 kDa) or beta-actin (42 kDa) as loading control. *Right*: Quantification of total tyrosine phosphorylation normalized to loading control (Ctrl), relative (rel) to DPI-treated or FL-stimulated wt is shown. Values are given as mean ± standard deviation, n = 3 – 4. Statistics: two-way ANOVA and Dunnett’s multiple comparisons test; * p ≤ 0.05; ** p ≤ 0.01 compared to respective wt. *Left:* Representative immunoblots are shown. Position of molecular weight standard bands from 35 – 180 kDa are indicated by dashes left and right of blots. 79 – G979L; 83 – G983L; 87 – G987L.
