# Supplementary figures and images for: Disrupting PTPRJ transmembrane-mediated oligomerization counteracts oncogenic receptor tyrosine kinase FLT3 ITD
Source: Front Oncol. 2022 Nov 14;12:1017947. doi: 10.3389/fonc.2022.1017947 (PMC9701752; doi:10.3389/fonc.2022.1017947)

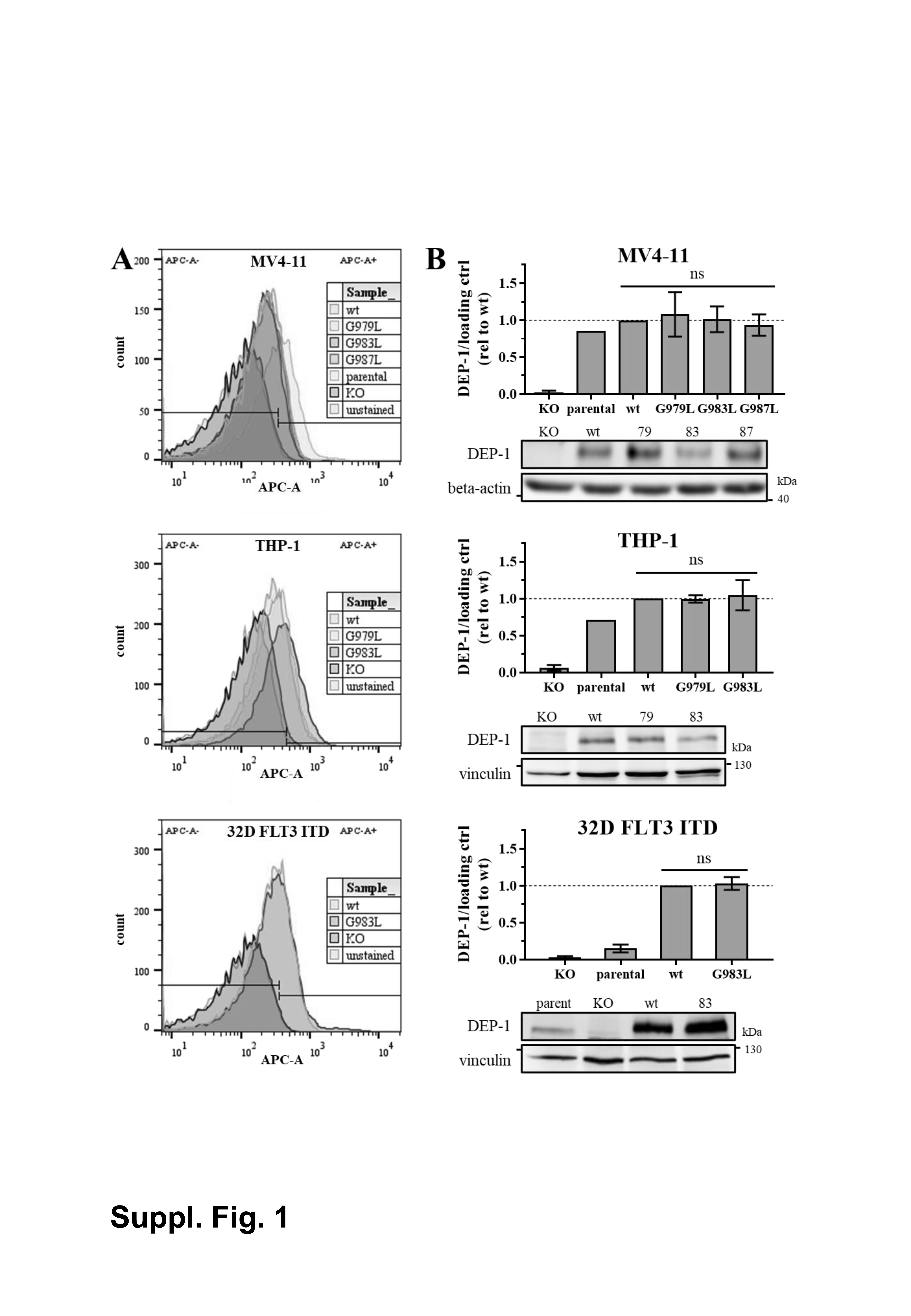

Supplement: Supplementary Figure 1 — Stable re-expression of PTPRJ in AML cell lines. PTPRJ wt and G-to-L variants were stably expressed in MV4-11, THP-1, and 32D FLT3 ITD PTPRJ KO cells by retroviral transduction. Cells were sorted for similar PTPRJ surface expression levels using flow cytometric based on PTPRJ detection. (A) PTPRJ surface expression. Cells were stained with APC-coupled α-CD148 (PTPRJ) antibody, and the APC signal was measured flow cytometrically. Histograms show cell-bound APC fluorescence intensity (APC-A). (B) Cellular PTPRJ expression level. Cell lysates were separated by SDS-PAGE, blotted, and analyzed with antibodies recognizing PTPRJ. For loading control, antibodies recognizing vinculin (124 kDa) and beta-actin (42 kDa) or Ponceau staining (not shown) were used. Graphs show PTPRJ signal normalized to loading control (ctrl), relative (rel) to respective wt. Values are given as mean ± standard deviation, with n = 5 – 6 for MV4-11 and n = 3 for THP-1 and 32D, except for MV4-11 and THP-1 parental n = 1. Statistics: one-way ANOVA and Dunnett’s multiple comparisons test; * p ≤ 0.05 compared to respective wt. ns – not significant. Representative immunoblots are shown below graphs. Positions of 40 and 130 kDa bands of molecular weight standard are indicated. 79 – G979L; 83 – G983L; 87 – G987L. [file Image_1.jpeg]

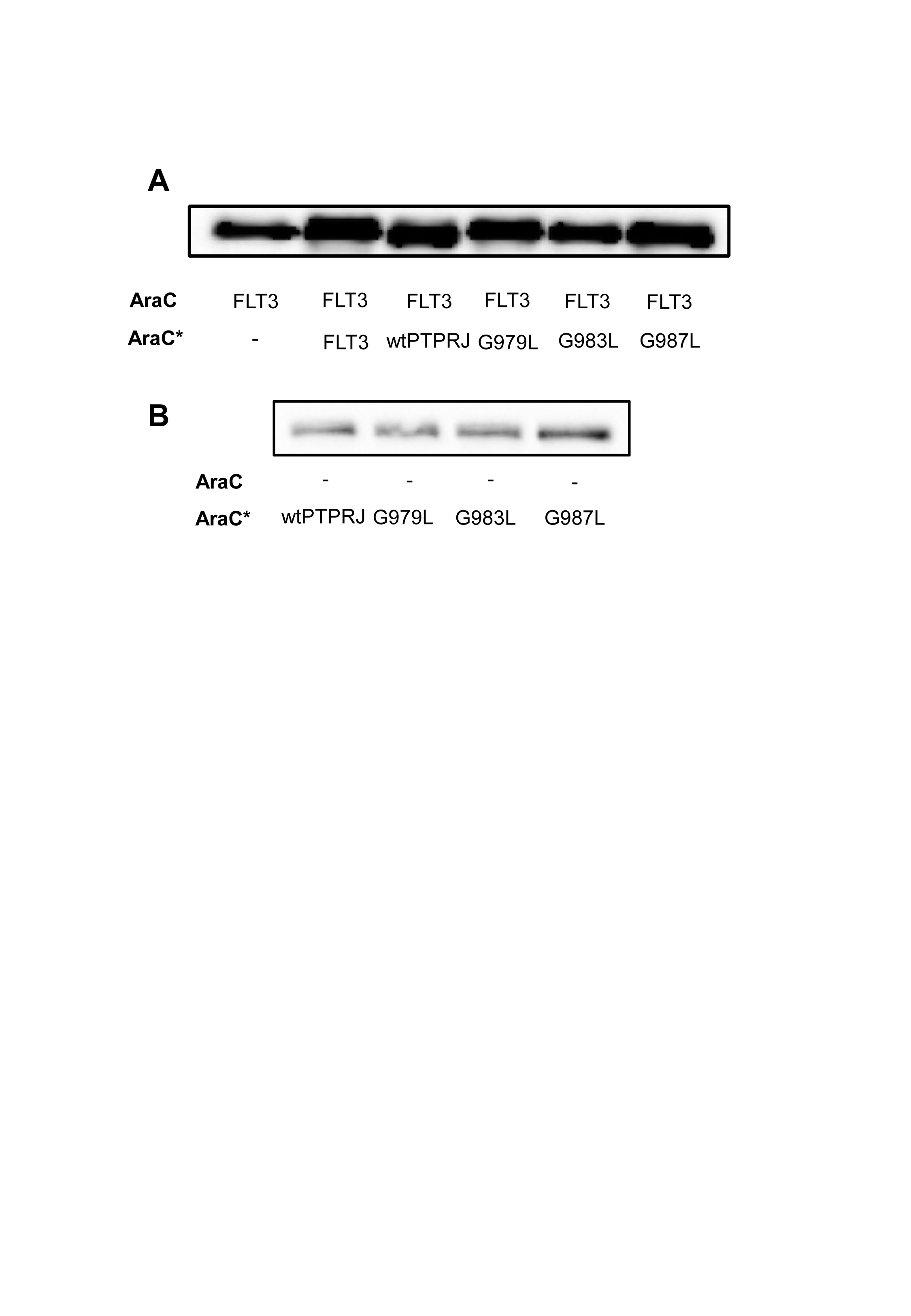

Supplement: Supplementary Figure 2 — Representative immunoblot analysis against MBP used to normalize the DN-AraTM assay results. Bacteria are always transformed with both AraC* and AraC-containing plasmids. The dash (-) represents an empty vector without the TMD construct of interest. [file Image_2.jpeg]

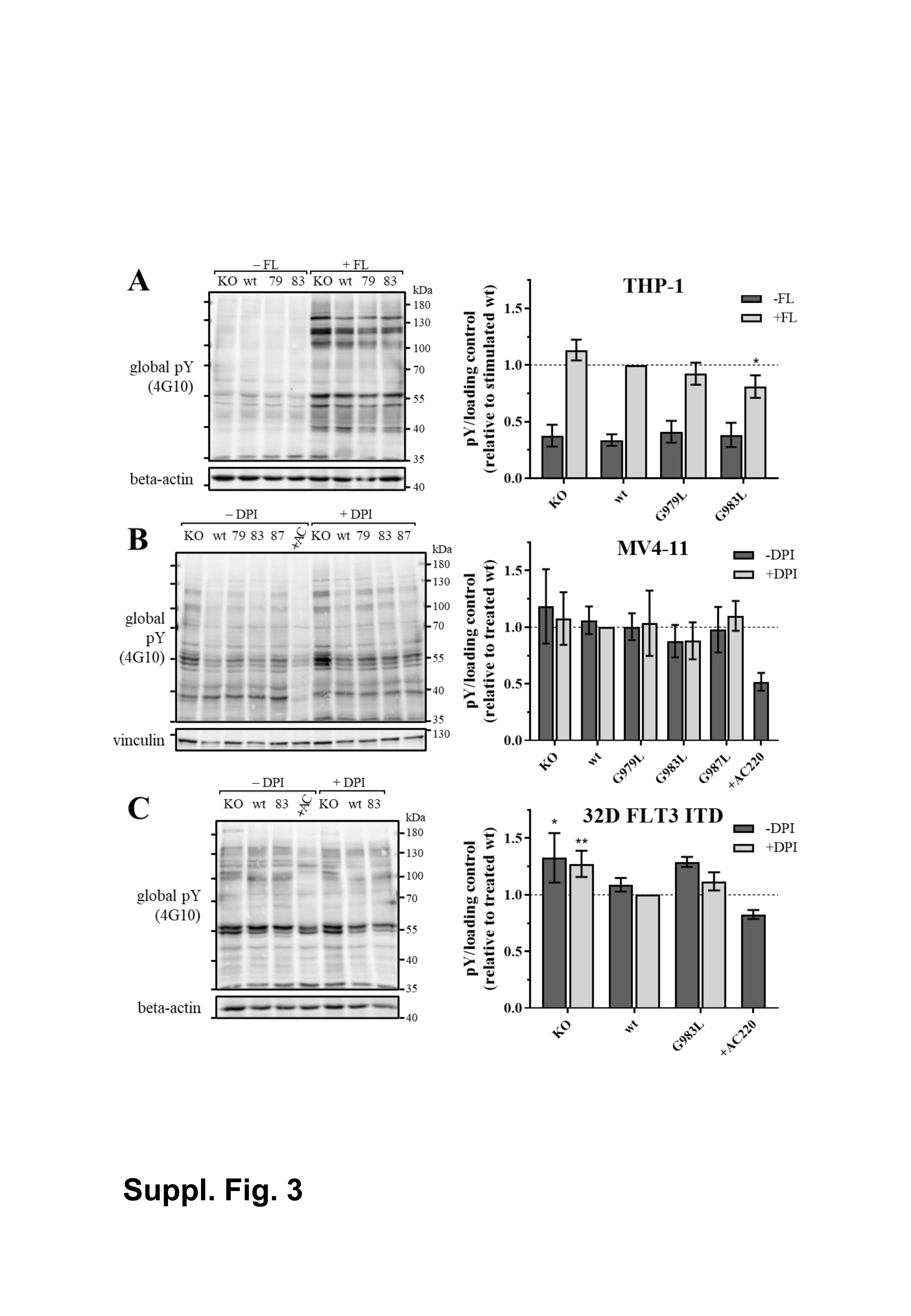

Supplement: Supplementary Figure 3 — Global protein tyrosine phosphorylation pattern of DEP-1 wt and TMD mutant expressing cell lines. THP-1 (A), MV4-11 (B), and 32D FLT3 ITD (C) PTPRJ KO cells or cells re-expressing DEP-1 wt, G979L, G983L, and G987L were starved for 4 h in serum- and cytokine-free medium and lysed in RIPA buffer. During starvation, MV4-11 and 32D FLT3 ITD cells were treated with DPI (0.5 µM), AC220 (+AC, 20 nM), or mock (DMSO), as indicated. THP-1 cells were stimulated with FL (200 ng/ml, 5 min) before harvest. Equivalent amounts of protein samples (15 – 20 µg) were separated by SDS-PAGE, blotted to a nitrocellulose membrane, and analyzed for global tyrosine phosphorylation using α-pY antibody (4G10). Blots were re-probed for vinculin (124 kDa) or beta-actin (42 kDa) as loading control. Right: Quantification of total tyrosine phosphorylation normalized to loading control (Ctrl), relative (rel) to DPI-treated or FL-stimulated wt is shown. Values are given as mean ± standard deviation, n = 3 – 4. Statistics: two-way ANOVA and Dunnett’s multiple comparisons test; * p ≤ 0.05; ** p ≤ 0.01 compared to respective wt. Left: Representative immunoblots are shown. Position of molecular weight standard bands from 35 – 180 kDa are indicated by dashes left and right of blots. 79 – G979L; 83 – G983L; 87 – G987L. [file Image_3.jpeg]
